# Recurrent HELLP Syndrome at 22 Weeks of Gestation

**DOI:** 10.1155/2017/9845637

**Published:** 2017-08-08

**Authors:** Peter Kascak, Milos Paskala, Peter Antal, Radovan Gajdosik

**Affiliations:** ^1^Department of Obstetrics and Gynecology, Faculty Hospital Trencin, Trencin, Slovakia; ^2^Faculty of Health, Alexander Dubcek University of Trencin, Trencin, Slovakia

## Abstract

We describe a case of a woman who presented with HELLP syndrome in two subsequent pregnancies (age 36 and 40), at gestational age of 22 weeks in both pregnancies with a rapid onset and progression. She presented with characteristic clinical symptoms and laboratory findings. Both pregnancies were delivered by caesarean sections due to deterioration in the mother's condition, although neither pregnancy progressed into the 1st stage of the syndrome according to Mississippi classification. This case highlights the admittedly rare but serious condition of recurring HELLP syndrome at a gestational age before the limit of viability of the fetus. In such situations, pregnancy termination should be considered to minimise risk to the mother's life and health.

## 1. Introduction

HELLP syndrome (Hemolysis, Elevated Liver enzymes, Low Platelet count) is a rare and serious pregnancy complication associated with a high risk of perinatal and maternal morbidity and mortality. Approximately 10% of HELLP syndrome cases occur at gestational age (GA) <27 weeks [1]. Some clinicians consider HELLP syndrome to be a severe form of preeclampsia, while others regard it a separate condition with characteristic clinical symptoms: headache, epigastric pain, nausea, vomiting, and visual disturbance in combination with laboratory findings of haemolysis, liver enzymes elevation, and thrombocytopenia [2]. It is important to differentiate HELLP syndrome from other thrombotic microangiopathies, especially in early pregnancy [3]. Clinical presentation may range from asymptomatic forms, such as nonspecific flu-like symptoms, to fully developed characteristic clinical signs [4]. A presentation of ascites as the first symptom of fulminant postpartum HELLP syndrome is rare [5]. Multiparity, twin pregnancy, chronic hypertension, obesity, diabetes, history of preeclampsia, and history of HELLP syndrome are considered as risk factors. The risk of HELLP syndrome recurrence is low at 3–5%, increasing with previous disease onset in early pregnancy [6]. HELLP is also associated with factor V Leiden mutation and the presence of anticardiolipin antibodies [4]. The severity of HELLP syndrome is commonly assessed according to the Mississippi classification and based on laboratory tests [1]. Early delivery or termination of pregnancy in extremely early forms of the disease is the recommended course of treatment. The choice of labor induction or, in most cases, urgent caesarean section, is based on fetal status, laboratory and clinical parameters, and cervical ripening [2, 4].

## 2. First Occurrence: 2009

A 36-year-old primigravida was admitted in our department at GA 21 + 3 with preeclampsia and HELLP syndrome. She complained of nausea and epigastric pain. At admission, she was normotensive and her laboratory findings showed blood count and hemocoagulation within normal ranges. Her biochemical analysis showed mild elevation in alanine aminotransferase (ALT) 0.96 *µ*kat/l (upper limit of normal (ULN), 0.57 *µ*kat/l) and aspartate aminotransferase (AST) 1.44 *µ*kat/l (ULN 0.53 *µ*kat/l). Quantitative proteinuria was 2.11 g/day. Her condition suddenly worsened two days after admission, with severe epigastric pain, nausea, vomiting, and blood pressure (BP) of 220/110 mmHg. Anticonvulsive therapy with magnesium, intravenous (IV) antihypertensive therapy (urapidil), and low molecular weight heparin (LMWH) was initiated. While her BP stabilised at 150/80 mmHg, her laboratory findings six and twelve hours after treatment administration showed rapid deterioration. Her platelet count decreased from normal levels to 58 × 10^9^/l, haemoglobin declined to 13 g/l, and ALT and AST increased to 30 and 80 times the ULN, respectively. Blood electrolytes and renal function parameters were within normal range. D-dimer level was elevated at 39.6 mg/l (ULN 0.47 mg/l), antithrombin III had decreased to 71.4%, and INR was 1.47 with prolonged thrombin time at 25 s. Lactate dehydrogenase (LD) was elevated at 94.73 *µ*kat/l (ULN 7.7 *µ*kat/l). Peripheral blood smear showed schistocytes. Haptoglobin level had decreased to 0.06 g/l. Based on these findings, its rapid deterioration, and presence of severe maternal symptoms (headache, vomiting), immediate termination of pregnancy was indicated due to the threat to mother's life and health. As her cervix score was 0 and her condition deteriorated rapidly, we opted for caesarean section. The female fetus weighed 280 g. We administered 1 g of fibrinogen and 1000 IU of AT III during surgery. Early postoperative course was uncomplicated and she recovered fast. Corticosteroids were not given. The patient's aminotransferase levels decreased gradually, there was no further worsening of thrombocytopenia, and all other hemocoagulation parameters improved to normal levels. Interestingly, urine protein after the termination increased to 5.3 g/day but decreased to normal level few days later. The patient was discharged eight days after the termination. She continued on antihypertensive, LMWH therapy, and bromocriptine for lactation cessation, and she was referred for a thrombophilic screen.

## 3. Second Occurrence: 2013

The same patient, at this point a 40-year-old gravida 2, para 0, was admitted to our unit at GA 21 + 2. She complained of epigastric pain for four hours without lateralisation, headache, chills, and continuous vomiting. However, she had neither diarrhoea nor urinary problems. After the pregnancy termination in 2009 she was examined by a haematologist and heterozygous factor V Leiden mutation was confirmed. At that point she was also prescribed antihypertensive treatment, which was stopped in 2013 at GA of 12 weeks; the patient was normotensive. From the beginning of this second pregnancy, prophylactic LMWH was administered by hematologist. A physical examination at admission revealed hypertension (200/100 mmHg) and proteinuria (+++). Her abdomen was tender in the epigastrium and the right hypochondrium by palpation, with no signs of peritoneal irritation. There was no uterine tenderness and the uterus was normotonic. Transabdominal ultrasound showed a live fetus with normal biometry (20 + 5) and an estimated fetal weight of 380 g. Initial laboratory tests showed normal haemoglobin (129 g/l) and platelet count (258 × 10^9^/l). Transaminases were elevated, ALT 9.33 *µ*kat/l and AST 15.06 *µ*kat/l. The evaluation of coagulation parameters showed positive D-dimer (5.2 mg/l); other parameters were normal. LD level was elevated at 17.17 *µ*kat/l. Other biochemical parameters were within normal range. IV anticonvulsive therapy with magnesium, antihypertensive therapy (nifedipine), and IV antiemetic (metoclopramide) were administered. Her subjective condition improved and BP normalised at 130/90 mmHg. Five hours after admission laboratory tests showed a decrease in haemoglobin and platelet count to 115 g/l and 146 × 10^9^/l, respectively, an increase in transaminase levels (ALT 16.1 *µ*kat/l, AST 34.38 *µ*kat/l, LD 26.67 *µ*kat/l) and D-dimer (19.97 mg/l), and a decrease in fibrinogen (from 3.0 to 2.4 g/l). Despite the temporary subjective improvement, her epigastric pain worsened and BP rose to 150/100 mmHg. Due to this recurrence of early-onset severe HELLP syndrome, a termination of pregnancy was indicated. Due to previous scar on uterus, unfavorable cervix score (CS 0) with respect to her condition, caesarean section was indicated. A nonviable male fetus weighing 455 g was delivered. Carbetocin and prostaglandins were administered during surgery due to uterine hypotonia. The postoperative course was uncomplicated. Corticosteroid therapy was not indicated. The patient was without any subjective complaints after the surgery. Another mild decrease in platelet count occurred after the termination, followed by an increase to normal levels and a decrease in haemoglobin level. Coagulation parameters showed persistent elevation of D-dimer and decrease in AT III to 59% with subsequent gradual improvement. Serum transaminase levels normalised gradually. Since this termination, the patient has not become pregnant again and has no children.

## 4. Discussion

We describe a rare but possible recurrence of preeclampsia and HELLP syndrome, in both instances at an early gestational age. In the analysis of 34 pregnancies with HELLP syndrome from Ostrava, the Czech Republic, no occurrence of HELLP syndrome earlier than GA of 28 weeks was recorded [7]. A ten-year analysis of pregnancies in a tertiary maternity centre in the Netherlands registered 26 cases of preeclampsia at GA < 24 weeks, 65% of which suffered serious maternal morbidity (16 with HELLP, five with eclampsia, four with pulmonary oedema, and one maternal death) [8]. In another study of 35,937 deliveries from United States, preeclampsia was diagnosed in 3,800 women, but only 39 cases (1%) occurred earlier than GA of 25 weeks [9]. All 39 women were treated for severe preeclampsia; five women had a placental abruption, nine had HELLP syndrome, five had acute renal insufficiency, and three had eclampsia. Twenty-two children (55%) were born alive, but only four (10%) survived, all severely physically or mentally disabled. A large retrospective analysis of 238,448 deliveries in all ten maternity centres in the Netherlands during a 14-year period described 161 pregnancy terminations due to severe hypertension (0.07% of deliveries) with a nonviable fetus based on early GA [10]. 96 mothers (60%) simultaneously developed HELLP syndrome; ten mothers suffered eclampsia. Among the 161 women, there were no maternal deaths and all deliveries were induced, contrasting with the recommended urgent pregnancy termination. Only one acute caesarean section delivery was described in this group in a patient with a uterine rupture and hypovolemic shock after prostaglandin induction. Perinatal mortality was 99.4%, meaning that only one child, born at GA of 25 weeks with a birthweight of 600 g, survived.

In the case we describe, the diagnosis of recurring of severe HELLP syndrome was confirmed in one patient in both her pregnancies. In the literature, we found only one similar case [6]. The patient was obese with hypertension and diabetes and developed severe HELLP syndrome simultaneously with severe superimposed preeclampsia in two consecutive pregnancies at the ages of 42 and 45 years at GA of 18 and 22 weeks, respectively. She was anticardiolipin antibody-positive. The higher age of this patient is consistent with our case and another case of a 41-year-old primigravida with severe HELLP syndrome at GA of 17 weeks [11].

Prevention of developing serious maternal morbidity and mortality is one of the main goals of treating HELLP syndrome [2, 12]. Of course, in nonviable fetus, the method of termination of pregnancy should be preferably vaginal, but other factors must be taken into account, such as cervix score, speed of progression of the disease, severity of symptoms, and chance to deliver/abort early. In our case, we opted for the expedited delivery by caesarean section as the chances of quick vaginal termination were very low. We think that prompt intervention in first pregnancy prevented the progression of HELLP syndrome to its most severe form (1st stage of syndrome according to Mississippi classification) and from developing more serious maternal complications. Rapid worsening of laboratory findings and clinical symptoms is linked to higher maternal morbidity especially in early cases of HELLP syndrome [13, 14]. The indication of caesarean section in 2nd pregnancy was highly influenced by the previous surgery. Expectant observation (>48–72 hours) with corticosteroid therapy to induce lung maturity and control the disease itself could be considered in cases when GA was approaching the fetal viability threshold [15]. Prolongation of the pregnancy might improve the neonatal outcome but can lead to intrauterine death or asphyxia and increase maternal morbidity. In a case report of severe HELLP syndrome at GA 21 + 0 by Merz and Gembruch, the disease was stabilised, but at GA 22 + 3 severe preeclampsia developed again and intrauterine fetal death was diagnosed [16]. In a cohort of 1,677 women at GA 24–34 weeks it was recommended to consider conservative management in a strictly selected subgroup, leading to significantly better neonatal and maternal outcomes [15]. However, in a group of women at GA < 24 weeks from the same study, conservative management did not improve the prognosis of the fetus while it greatly increased maternal morbidity. Based on this evidence, we did not consider conservative management in either syndrome presentation of our patient. The suggestion that HELLP syndrome at GA < 26 weeks occurs only in women with superposed preeclampsia or with positive antiphospholipid antibodies can be contested [17]. In our case, we ruled out antiphospholipid syndrome and, in her first pregnancy, our patient was not hypertensive before disease onset. However, after termination we confirmed heterozygous factor V Leiden mutation. Corticosteroid therapy in HELLP syndrome is still a matter of intensive research; however, published data clearly suggest a benefit [1, 18]. We did not administer steroid treatment to our patient, but, given this increasing evidence base, we have incorporated steroid treatment into our departmental guidelines. Withdraw of antihypertensive therapy and prescription of low dose aspirin in her 2nd pregnancy in an outpatient way are both certainly questionable.

## 5. Conclusion

The occurrence of HELLP syndrome at an early gestational age below the fetus viability level is an extremely rare condition, but obstetricians must be vigilant about it because this rapidly progressing disease might endanger a mother's life. Therefore, a timely diagnosis and pregnancy termination is essential.
